# The Association of Demographic Characteristics and Food Choice Motives with the Consumption of Functional Foods in Emerging Adults

**DOI:** 10.3390/nu12092582

**Published:** 2020-08-25

**Authors:** Lieke Vorage, Nicola Wiseman, Joana Graca, Neil Harris

**Affiliations:** 1 School of Medicine, Griffith University, Gold Coast Campus, Southport, QLD 4222, Australia; nicola.wiseman@griffith.edu.au (N.W.); n.harris@griffith.edu.au (N.H.); 2 Morlife (Pty) Ltd., Arundel, QLD 4214, Australia; marketresearch@morlife.com

**Keywords:** functional foods, consumption, attitude, motives, emerging adults

## Abstract

The functional food market is one of the fastest growing segments of the global food industry. The aims of this study were to understand the association of demographic characteristics and food choice motives (FCMs) with (a) attitudes toward functional foods and (b) consumption of functional foods in Australian emerging adults. Data were collected through a paper-based and online questionnaire completed by 370 young adults aged between 17 and 29 years. A binomial logistic regression was used to determine the association between demographic characteristics and FCMs with attitudes towards functional foods. The logistic regression model was statistically significant at χ^2^(11) = 48.310 (*p* < 0.001) and explained 18.1% of the variance in attitude towards functional food. Of the several predictors, only the FCMs natural content and weight control were statistically significant. A binomial logistic regression was also used to determine the association between demographic characteristics and FCMs with the consumption of functional foods. The logistic regression model was statistically significant at χ^2^(9) = 37.499 (*p* < 0.001) and explained 14.1% of the variance in functional food consumption. Of the eight predictors, three were statistically significant: living situation, natural content and health. Findings highlight that when targeting emerging adults, functional food companies could benefit from promoting the natural and health properties of their products. Furthermore, consumption can be increased by targeting the parents of emerging adults and by designing functional foods that attract emerging adults interested in controlling weight.

## 1. Introduction

The term ‘functional foods’ was first introduced in 1984 in Japan [1]. Although there are a number of ways to define the term ‘functional food’ [2], to date, there is no universally accepted definition for this group of foods [3]. Several national authorities and scientific organizations have formulated definitions [4]. The International Food Information Council defines functional foods as ‘*foods that may provide health benefits beyond basic nutrition’*. [5]. The American Dietetic Association defines functional foods as ‘*foods that provide additional health benefits that may reduce disease risk and/or promote optimal health’* [6] The EU Project ‘Functional Food Science in Europe’ specifies functional foods as ‘*foods that are satisfactorily demonstrated to affect beneficially one or more target functions in the body, beyond adequate nutritional effects, in a way that is relevant to either an improved state of health and well-being and/or reduction of risk of disease*’. [7] As this study was conducted in Australia, in this paper, the working definition of the National Centre of Excellence in Functional Foods in Australia will be used, describing functional foods as “*foods that meet consumer needs for general health and wellbeing, and the prevention and management of compromised health conditions*” [8].

In 2017, the sale of functional foods was valued at US$247 billion [9] and it is expected that the market will continue to grow [10]. Several factors, such as the increased awareness of the connection between nutrition and health [1], the rise in chronic diseases [11] and the aging population, have contributed to the growing demand for functional foods [12]. A growing body of research suggests that certain functional foods are effective in decreasing morbidity and increasing quality of life within the population [13,14,15,16,17].

When selecting food products, people take into account certain motives that govern their food choice [18]. Previous studies have investigated the effect of these food choice motives (FCMs) on eating behaviour. An increased emphasis on the motives of health, natural content and weight control has been found to associate with healthy eating behaviour [19,20,21]. The motive of convenience has been shown to associate with functional food consumption [22], the higher the importance of convenience, the heavier the use of functional foods. Last, the motive of familiarity has been found to negatively correlate with functional food consumption [22]. Functional foods have a novelty aspect to them, due to their functionality which is not overtly recognised in conventional food products [23]. The average consumer tends to reject too much novelty in food [24]. However, functional food consumers seem to be motivated to try unfamiliar foods [23].

Some demographic variables have been found to influence functional food consumption. In general, females are more likely to consume functional foods compared to males [25,26,27]. Studies focusing on attitudes suggest that females are also more positive about functional foods [28,29,30,31]. Another relevant demographic characteristic is the presence of children in the household [32], with parents more likely to purchase functional food products [33], as they are looking for wholesome foods that lay a strong foundation for the health of their children [34]. Further, a number of studies have indicated a relationship between functional food consumption and income [25,26], with higher socio-economic groups showing an increased willingness or ability to pay premium prices for functional foods [35].

Emerging adults have been identified in the literature as an interesting target group for functional foods because of their willingness to accept novel products [36,37] and their positive attitude towards high-technology food processing [38]. Furthermore, research has shown that emerging adults are more health conscious than older generations and more willing to pay a premium price for functional foods that reduce disease risk or promote good health [39]. Emerging adulthood is defined as the life stage between adolescence and young adulthood [40]. From 1970 onwards, the transition into adulthood in high-income countries has become longer. Traditional milestones of adulthood, such as having a stable job, marriage and parenthood, are routinely delayed to late twenties or early thirties [41,42]. Therefore, emerging adulthood is now regularly defined as lasting approximately from age 18 to approximately age 29 [40]. As a life stage, emerging adulthood is a time where individuals are establishing lifestyle habits, including dietary practices [43]. This makes this population of particular interest as a target group for functional foods

The influence of demographic variables on attitudes toward and consumption of functional foods has been reported in several articles [25,26,27,28,29,30,31,32,33,34]. However, limited research has sought to understand the links between demographic characteristics and food choice motives (FCMs) underlying functional food consumption [22]. Recognising that attitudes towards functional food products can be location, population and culture specific [37,44], even among Western countries [45,46], it is important to know more about the drivers of functional food consumption in rapidly growing emerging markets, such as Australia. The aims of the present work were to understand the association of demographic characteristics and FCMs with (a) attitudes toward functional foods and (b) consumption of functional foods in Australian emerging adults.

## 2. Materials and Methods

### 2.1. Participants and Design

The study followed a cross-sectional design. Ethical approval was obtained from the Griffith University Human Research Ethics committee (GU Ref No: 2017/166) on the 20th of March 2017. To ensure valid results, a sample size of 350 emerging adults was determined appropriate, assuming a medium effect (standardized difference of 0.3). This study used convenience sampling to collect data. Data were collected in April 2017 through a paper-based and online questionnaire of 370 emerging adults between the ages 17 and 29 years. While the contemporary age grouping for emerging adults is 18 to 29 years, the human research ethics committee required the sampling frame to be inclusive of 17 year olds, given the university population and the university setting. The paper-based questionnaire was distributed to students at the Griffith University Gold Coast Campus, which is a large comprehensive campus with approximately 10,000 undergraduate students. Participants were randomly approached in person at common spaces of the campus at various times during weekdays. The online questionnaire was distributed to Griffith University students in the ‘Volunteer for Important Research’ e-mail from Griffith University. Some information about the study and a link to the online survey were put in this e-mail. The first page of the questionnaire contained an information sheet describing the research and consent was given by completion of the survey. It took participants an average of 10 minutes to fill in the questionnaire. The questionnaire can be found in the Supplementary Materials.

### 2.2. Food Choice Motives

To measure FCMs, three scales were used. The Food Choice Questionnaire (FCQ), developed by Steptoe, Pollard and Wardle [18], was used to measure eight dimensions of FCMs including health, natural content, weight control, sensory appeal, convenience, price, familiarity and mood. The FCQ has been applied in a number of studies and has been used in a student population [18,19]. The scale has been found to be reliable and internally consistent [18,19]. Ethical dimensions of food choice motives including ecological welfare, political values and religion were measured using scales developed and validated by Lindeman and Väänänen [47]. The food choice motive of fitness was measured using a scale developed by Lockie, Lyons, Lawrence and Mummery [48]. In total, twelve dimensions of food choice motives were measured in this study. The FCQ started with the sentence ‘*It is important to me that the food I eat on a typical day…*’ and was followed by a series of statements covering the twelve dimensions measured by the FCQ. The participants were asked to select an appropriate response for each statement ranging from (1) ‘*not at all important*’ to (4) ‘*very important*’. The score of each food choice motive was calculated by averaging the unweighted rating for the individual items of the scale, so it could range from 1 to a maximum of 4.

### 2.3. Attitudes Towards Functional Food and Functional Food Consumption

Before measuring attitudes toward and the consumption of functional foods, respondents were given the following definition of functional food: ‘*foods that meet consumer needs for general health and wellbeing, and the prevention and management of compromised health conditions’* [8]. The definition was supported by several examples such as *‘margarine which can lower cholesterol’* and *‘a yoghurt drink with probiotics’*. Attitudes toward functional foods was measured using a scale adapted by Marina, Marija, and Ida [49]. Respondents were asked to express their agreement with seven different statements on a 5 point Likert scale that ranged from (1) ‘*completely disagree*’ to (5) ‘*completely agree*’. The overall attitude score was calculated by averaging the unweighted ratings for the seven individual items, so the score could range from 1 to 5. Functional food consumption was measured by asking respondents ‘*How often do you consume functional foods?*’ Respondents could indicate their functional food consumption frequency on a scale ranging from (1) ‘*every day*’ to (10) ‘*I do not consume functional foods*’. Standard demographic information was collected, including gender, age, year of enrolment, living arrangement, responsibility for food shopping, marital status and income.

### 2.4. Statistical Analysis

Cronbach’s alphas were calculated for the twelve FCMs and the attitude scale. If not reported otherwise, the Cronbach’s alpha is above 0.7. Shapiro–Wilk tests indicated that the data were not normally distributed. To evaluate the differences in importance of the motives by demographic characteristics Mann–Whitney U tests were performed. As the distributions of demographic variables were not equal, mean ranks were used instead of medians. A binomial logistic regression was performed to ascertain the effects of demographic characteristics and the motives underlying food choice on the attitudes toward and the consumption of functional food. Results were considered statistically significant at <0.05. Analyses were conducted using SPSS version 23 (IBM Corporation, New York, NY, USA).

## 3. Results

### 3.1. Sample

Table 1 describes the characteristics of the participants by gender, age, year of enrolment, living situation, responsibility for food shopping, marital status and personal annual income.

### 3.2. Demographic Characteristics and Food Choice Motives

Table 2 shows the median scores for each FCM for all participants (Table 2). The scores of the different FCMs all ranged between a minimum value of 1 and a maximum value of 4.

To investigate whether the four key demographic variables of gender, age, living situation and income were associated with the motives underlying the selection of food, Mann–Whitney U tests were performed. These analyses are presented in Table 3. First, analyses were conducted to determine whether there were significant differences in FCMs between males and females. The mean rank scores for natural content (*p* = 0.018), ecological welfare (*p* < 0.001) and weight control (*p* = 0.026) were significantly higher in females. Interestingly, males had a higher mean rank score in religion compared to females (*p* = 0.035). Participants were divided into two age categories—young emerging adults (17–20) and older emerging adults (21–29)—based on the age distribution in the sample. This and all other recodes can be found in the Supplementary Materials. Younger students had a significantly higher mean rank score for the motive of weight control than older students (*p* = 0.036). Respondents who lived independently had a higher mean rank score in the motive health than those who lived dependently (*p* = 0.021). No significant associations were found between the motives and income (all *p* values > 0.05).

### 3.3. Association of Demographic Characteristics and Food Choice Motives with Attitudes

To determine the association between demographic characteristics and FCMs with participant attitudes toward functional foods, a binomial logistic regression was performed. Key demographic variables and FCMs that were significantly correlated with attitudes toward functional food were included in the model. A Spearman’s correlation test showed that age and the FCMs natural content, health, political values, mood, ecological welfare, familiarity, weight control, fitness and religion were significantly correlated with attitudes. Scores of each motive were recoded into two groups (namely a low score group and a high score group), by using the median score to create similar sized groups. Using the same procedure, attitudes was recoded into two groups, including a negative attitude and a positive attitude group.

The logistic regression model was statistically significant at χ^2^(11) = 48.310 (*p* < 0.001). The model explained 18.1% (Nagelkerke R^2^) of the variance in attitude towards functional food and correctly classified 68.1% of cases. Of the nine predictors, only the FCMs natural content and weight control were statistically significant (Table 4). Participants who had a high score in the motive natural content were 1.82-fold more likely to have a positive attitude towards functional foods. Participants who had a high score in the motive of weight control were 2.79-fold more likely to have a positive attitude towards functional foods.

### 3.4. Association of Demographic Characteristics and Food Choice Motives with Consumption

To determine the association between demographic factors and FCMs with the consumption of functional food, a binomial logistic regression was performed. Key demographic characteristics and FCMs that were significantly correlated with consumption of functional food were included in the model. A Spearman’s correlation test showed that participants’ living situation and the motives natural content, health, ecological welfare, weight control and fitness were significantly correlated with the consumption of functional food. Again, scores of each motive were recoded into two groups, a low score group and a high score group. The variable functional food consumption was also recoded into two groups including a low and a high consumption group.

The logistic regression model was statistically significant at χ^2^(9) = 37.499 (*p* < 0.001) (Table 5). The model explained 14.1% (Nagelkerke R^2^) of the variance in functional food consumption and correctly classified 68.9% of cases. Of the eight predictors, only three were statistically significant, these included living situation, natural content and health (Table 5). Participants who lived dependently were 2.79-fold more likely to have a high functional food consumption than emerging adults who lived independently. Participants who had a high score on natural content were 2.05-fold more likely to have a high functional food consumption than emerging adults who had a low score on natural content. Finally, those who had a high score for health were 1.80-fold more likely to have a high functional food consumption than emerging adults who had a low score on health. All statistical output can be found in the Supplementary Materials.

## 4. Discussion

The complementary face-to-face and online participant recruitment strategies yielded a total sample of 370. All participants were attending university courses. While university students are not a truly representative sample of the emerging adult population, it is worth noting that in Australia approximately two-thirds of the emerging adults who are currently studying to obtain non-school qualifications, are enrolled at university [50]. Further, females were over represented in the sample drawn (73.5%, *n* = 272 of 370). This can be explained, as more females than males in this age group attend higher education [51], the topic could have been of greater interest to females due to the fact that females are generally more interested in nutrition [52] and have a more positive attitude towards functional foods [28,29,30,31] and males are less willing to participate in survey based research [53].

### 4.1. Food Choice Motives

Emerging adults indicated that price, sensory appeal, convenience and health were strong motives contributing to their food choices. This is comparable with research conducted by Steptoe et al. [18] and Carrillo, Varela, Salvador and Fiszman [54]. These authors found that sensory appeal, convenience, price and health are considered the most important motives. Young adulthood is a time in which many individuals move away from home and become responsible for their own food choices [55]. In this life stage, emerging adults gain control over their own finances and food prices begin to have a stronger influence on food choice [55]. Young adults also experience many new barriers to living a healthy lifestyle such as high stress, poor sleep and challenges in time management [43], which increases the need for convenience in food choice [56].

### 4.2. Associations Between Demographic Characteristics and Food Choice Motives

Several demographic characteristics were associated with the importance participants placed on different FCMs. Females had a significantly higher mean rank score in the motives of natural content, ecological welfare and weight control compared to men. Previous studies have related these FCMs to healthy eating behaviour [19,20,21]. There is strong evidence to suggest that the difference in the importance placed on naturalness, ecological welfare and weight control can explain why women generally eat healthier in comparison to men [19]. In contrast, men gave significantly higher ratings for the motive religion. Mixed results are found in the literature about the role of religion by gender in food choice. Share and Stewart-Knox [57] found that boys placed greater importance on religion in making food choices than girls but Lindeman and Väänänen [47] did not find any gender differences.

Age had a significant influence on the motive of weight control. Young emerging adults (aged 17–20) gave a higher rating for weight control than older emerging adults (aged 21–29). This finding contradicts existing literature [18,58], which has shown that weight control becomes more important with increasing age. However, these studies used study samples with a wide range of ages, instead of only young adults, which makes comparison difficult. Hong et al. [59] found that body image becomes more positive during young adulthood. Since a negative body image has shown to increase weight control behaviour in adolescents [60], the higher importance of weight control for young emerging adults in this study may be explained by lower body image.

Emerging adults who lived independently reported higher scores for the motive of health compared to emerging adults living dependently. Previous research has shown that young adults living in an apartment/house were more involved in food preparation than young people who lived with their parents or at campus housing [61]. This higher level of involvement possibly leads to a higher concern for health when choosing food, as previous studies have shown a positive relationship between food involvement and healthy eating patterns [62,63]. No effect of income on the importance of the motives was found, probably due to the fact that there was a very low variation in income in the study population.

### 4.3. Effect of Demographic Characteristics and Food Choice Motives on Attitudes toward and Consumption of Functional Foods

The two binomial logistic regression models performed in this study showed that demographic characteristics and FCMs are associated with attitudes towards and the consumption of functional foods. First, the motive natural content was able to predict attitudes towards and the consumption of functional foods. This finding supports previous research showing a positive relationship between the FCM natural content and healthy eating behaviour [19,20,64]. A general concern that functional foods could be perceived as less natural compared to conventional products has emerged in the literature [65,66] However, the results of the current study suggest that functional foods are not perceived as unnatural, as participants who value natural content were more likely to have a positive attitude towards and were more likely to have a high consumption of functional food.

A relationship between the motive weight control and having a positive attitude towards functional foods was found. This result is supported by previous research showing a relationship between an increased emphasis on weight control and healthy eating patterns [20]. However, weight control was not able to significantly predict functional food consumption. It appears that respondents who place a greater emphasis on weight control are attracted to functional foods, but this does not translate through to consumption. This may be caused by a misperception about functional food. Functional foods are usually not specifically focused on helping individuals to control their weight, but rather on enhancing health or reducing risk of disease through added nutrients [11]. However, since health and weight control are related concepts [18], consumers might think that functional foods do help with maintaining body weight. When it comes to actual purchase for consumption, individuals may recognise that these products are not oriented on weight control and, hence, do not match with their purchase criteria.

The FCM health predicted participants’ consumption of functional foods. This is in line with previous research, which shows a relationship between health, healthy eating behaviour and functional food consumption [19,20,22]. Existing literature indicates that health is one of the most important FCMs [18,54]. This was also reflected in our study findings, as almost no respondents indicated that health was ‘*not at all important*’ to them (3.4%). Interestingly, the motive health was not able to predict attitudes toward functional food. The low variance in the importance of health in our study population, may explain why health was not able to predict attitudes toward functional foods. Health was, however, able to explain the consumption of functional food which indicates that those who indicate that health is ‘*very important*’, compared to those who find health ‘*a little*’ or ‘*moderately important*’ are more likely to consume these products.

Brecic et al. [22] found a relationship between convenience and functional food consumption. However, in the current study, the FCM convenience did not influence participants’ attitude towards and the consumption of functional foods. Carrillo et al. [54] found that the motives health and convenience were not related to each other, and represent two distinct ways of reasoning, namely ‘*practical versus beneficial*’ when choosing food. The lack of association between convenience and the consumption of functional food in this study suggests that consumers do not perceive functional foods as an expedient means to healthy or functional eating. This is an interesting finding that suggests there is opportunity to promote this aspect of functional foods. Similarly, previous research has identified price as an important factor influencing food choice [67,68], with a number of different studies suggesting the premium price for functional foods as a major barrier to acceptance and buying intention [28]. However, no relationship between the FCM price and the attitude towards or the consumption of functional foods was found in this study. This result may indicate that for participants, increased cost may not be as important as other FCMs, such as the healthiness of the product.

The absence of a relationship between familiarity and functional food consumption may indicate that functional foods are no longer seen as novel by Australian consumers. Functional foods have been a popular food category since the early 1990, meaning that they have been around for almost 30 years [22]. The fact that Brecic et al. [22] did find a negative relationship between familiarity and functional food consumption can be attributed to the fact that the study was executed in Croatia, as Eastern Europe is one of the newer markets for functional foods [22].

No relationship was found between gender and the attitude towards and consumption of functional foods, although this was expected based on previous research [25,26,27,28,29,30,31]. According to Dagevos [69] the explanatory power of socio-demographic variables on food choice behaviour has been decreasing. Modern consumers defy traditional segmentation by age, gender or income [69]. Our research contributes to the pool of studies which also did not find a difference in the attitude towards functional foods [23] and consumption of functional food [70,71,72] between gender groups.

Finally, living situation was able to predict the consumption of functional foods, with those living dependently consuming more functional foods than those that lived independently. No differences were found in the attitude towards functional foods. This demonstrates that the consumption of functional foods in emerging adults living with their parents/grandparents is not higher because they are more interested in functional foods, but because their parents buy these products for them. Previous research has found that the presence of children increases the concern for diet enhancement in the household [32,73] and the demand for functional dairy products [33]. This study contributes to this line of research by showing that students living at home consume more functional foods than those living independently.

As the participants in this study that reported natural content and health as FCMs were more likely to consume functional foods, it may be beneficial for functional food companies to take these factors into account when creating products, as the higher the perceived naturalness and healthiness of the functional food, the more it will appeal to the functional food consumer. This study also showed that individuals who placed a greater emphasis on controlling their weight had a more positive attitude towards functional foods, making them an interesting target group. However, they did not have a higher consumption of functional foods, probably due to the misconception they had about this product category. To increase the consumption of functional foods in people concerned with maintaining weight, new functional food products could be designed which possess health benefits and are also low in calories, sugar and fat, thereby expanding the target group for functional food products. Lastly, as emerging adults living dependently had a higher consumption of functional foods compared to those living independently, one way to increase the consumption of functional foods in emerging adults may be to target parents to promote the benefits of these products.

Although this research offers interesting insight regarding the motives driving the consumption of functional foods by young adults, further research should be conducted to detect additional drivers and predictors of attitudes toward and consumption of functional foods. Furthermore, future research should address some of the limitations of this study, such as the overrepresentation of females and those enrolled in tertiary education in the study population.

## 5. Conclusions

This study found that demographic characteristics and FCMs are able to predict attitudes towards and the consumption of functional foods in emerging adults. Emerging adults who value the FCMs of natural content and weight control are more likely to have a positive attitude towards functional foods. Further, those who live dependently and put greater emphasis on the FCMs natural content and health are more likely to have a higher consumption of functional foods. The practical implications of this study for functional food companies could be to focus more on emphasizing the natural and healthful properties of their products and targeting parents of emerging adults to promote the benefits of these products. Finally, by designing new functional food products that attract young adults interested in controlling weight, the target group for functional foods could be expanded.

## Figures and Tables

**Table 1:** Demographic characteristics of the participants.

Demographic Factors		Total (*n*)	%
Gender	Male	98	26.5
	Female	272	73.5
Age	17–20	225	60.8
	21–29	145	39.1
Year of enrolment	1st	162	43.8
	2nd	82	22.2
	3rd	71	19.2
	4th	26	7.0
	Other	29	7.8
Living situation	Dependent	211	57.0
	Independent	159	43.0
Responsible for food shopping	Me	99	26.8
	Me and someone else	159	43.0
	Someone else	111	30.0
	Missing values	1	0.3
Marital status	Single	296	80.0
	Married/Partnership	71	19.2
	Separated/Divorced	2	0.5
	Missing values	1	0.3
Income	10,399 or less	145	39.2
	10,400–20,799	124	33.5
	20,800–31,199	46	12.4
	≥31,200	44	11.9
	Missing values	11	3.0

**Table 2:** Median score for FCMs.

Food Choice Motives	Median Score
Price	3.33
Sensory appeal	3.25
Convenience	3.00
Health	3.00
Fitness	3.00
Mood	2.83
Ecological welfare	2.80
Natural content	2.67
Weight control	2.67
Familiarity	2.33
Political values	2.25
Religion	1.00

**Table 3:** Differences in the mean rank of the FCMs by gender, age, living situation and income.

**Gender**				**Age (in Years)**			
**Motive**	**U**	**Mean Rank**		**Motive**	**U**	**Mean Rank**	
		**Females** **(*n* = 272)**	**Males** **(*n* = 98)**			**17–20** **(*n* = 225)**	**21–29** **(*n* = 145)**
Natural content	10,789.0 *	190.24	160.89	Natural content	14,420.5	176.05	192.36
Convenience	12,514.5	181.52	187.14	Convenience	15,864.5	183.39	182.41
Health	11,941.0	184.27	172.10	Health	14,426.0	175.87	188.91
Political Values	11,839.0	183.66	171.82	Political values	14,019.5	175.87	188.91
Mood	11,374.0	184.74	168.5	Mood	14,782.5	177.69	184.91
Ecological welfare	9,953.5 **	195.64	151.61	Ecological welfare	15,885.0	183.23	185.19
Sensory appeal	11,619.5	191.12	168.07	Sensory appeal	15,655.0	182.39	189.03
Familiarity	12,750.0	183.05	186.69	Familiarity	16,013.0	184.19	183.70
Price	12,704.0	185.12	180.83	Price	15,789.0	185.20	182.15
Weight control	11,157.5 *	191.83	164.03	Weight control	14,057.5 *	193.74	170.12
Fitness	12,450.0	182.27	194.46	Fitness	15,827.5	187.66	182.16
Religion	11,666.5 *	179.39	202.45	Religion	16,233.5	185.15	186.04
**Living Situation**				**Income**			
**Motive**	**U**	**Mean Rank**		**Motive**	**U**	**Mean Rank**	
		**Depen-dent** **(*n* = 211)**	**Indepen-dent** **(*n* = 159)**			**<$20.799** **(*n* = 269)**	**>$20.800** **(*n* = 90)**
Natural content	13,697.0	169.99	190.19	Natural content	10,865.5	174	186.03
Convenience	14,713.5	166.38	191.71	Convenience	10,975.5	174.42	186.68
Health	12,993.0 *	166.38	191.71	Health	10,529.5	172.04	187.85
Political values	14,144.0	172	182.78	Political values	10,769.5	172.95	185.12
Mood	14,720.0	178.04	174.32	Mood	11,205.5	174.27	177.20
Ecological welfare	15,310.0	181.75	177.57	Ecological welfare	11,647.5	177.62	182.13
Sensory appeal	15,467.5	179.15	183.57	Sensory appeal	10,791.0	174.76	193.60
Familiarity	14,816.5	175.73	185.88	Familiarity	11,166.0	175.48	187.43
Price	14,537.0	174.72	187.44	Price	10,809.5	182.51	166.46
Weight control	14,810.0	176.19	186.60	Weight control	11,664.0	178.59	184.21
Fitness	15,449.5	179.22	184.69	Fitness	11,726.0	178.59	184.21
Religion	15,389.5	178.94	185.08	Religion	15,361.0	183.12	170.68

U: Mann–Whitney U, ** (*p* ≤ 0.001),* (*p* ≤ 0.05)

**Table 4:** The association of demographic characteristics and FCMs with attitudes toward functional food.

	B	SE	Wald	Df	P	Odd Ratio	95% CI for Odds Ratio	
							Lower	Upper
Age	−0.06	0.04	2.44	1	0.118	0.94	0.87	1.01
Gender	−0.20	0.29	0.47	1	0.494	0.82	0.47	1.14
Natural content	0.60	0.30	3.91	1	0.048	1.82	1.01	3.29
Health	0.36	0.29	1.57	1	0.210	1.43	0.82	2.51
Political values	−0.10	0.29	0.12	1	0.733	0.91	0.52	1.60
Mood	0.07	0.27	0.06	1	0.810	1.07	0.63	1.82
Ecological welfare	0.26	0.30	0.78	1	0.379	1.30	0.73	2.32
Familiarity	0.19	0.25	0.59	1	0.441	1.21	0.74	1.97
Weight control	1.03	0.27	14.65	1	0.000	2.79	1.65	4.72
Fitness	−0.37	0.29	1.63	1	0.202	0.69	0.39	1.22
Religious	0.43	0.26	2.74	1	0.098	1.54	0.92	2.58
Constant	−2.38	1.09	4.74	1	0.029	0.09		

B: coefficient for the constant, SE: standard error, Wald: Wald chi-square test, Df: degrees of freedom, P: *p* value, and CI: confidence interval.

**Table 5:** The association of demographic characteristics and FCMs with functional food consumption.

	B	SE	Wald	Df	*P*	Odd Ratio	95% CI for Odds Ratio	
							Lower	Upper
Age	0.03	0.04	0.43	1	0.511	1.03	0.94	1.12
Gender	0.35	0.29	1.51	1	0.219	1.42	0.81	2.50
Living situation	1.03	0.28	13.50	1	0.000	2.79	1.62	4.83
Natural content	0.72	0.31	5.30	1	0.021	2.05	1.11	3.77
Health	0.59	0.30	3.95	1	0.047	1.80	1.01	3.21
Ecological welfare	0.13	0.28	0.25	1	0.620	0.87	0.50	1.50
Weight control	0.09	0.27	0.10	1	0.749	1.09	0.64	1.85
Fitness	0.26	0.29	0.78	1	0.377	1.30	0.73	2.31
Constant	3.99	1.35	8.70	1	0.003	0.02		

B: coefficient for the constant, SE: standard error, Wald: Wald chi-square test, Df: degrees of freedom, P: p value, and CI: confidence interval.

## Supplementary Materials

The following are available online at https://www.mdpi.com/2072-6643/12/9/2582/s1, Supplementary File S1: Emerging adult questionnaire; Supplementary File S2: Recoding; Supplementary File S3: Statistical output.
