# Supplementary material for: The Association of Demographic Characteristics and Food Choice Motives with the Consumption of Functional Foods in Emerging Adults
Source: Nutrients. 2020 Aug 25;12(9):2582. doi: 10.3390/nu12092582 (PMC7551355; doi:10.3390/nu12092582)
Supplement: Supplementary file 1 [file nutrients-12-02582-s001.zip › Supplements/Supplements_Nutrients_Appendix S1_Emerging_adult_questionnaire_Lieke_Vorage.docx]

## Appendix S1: Emerging adult questionnaire

**Participant Information Sheet**

**Understanding the motives underlying the attitude towards and consumption intention of functional foods and evaluating the brand image of the functional food company Morlife.**

**Who is conducting the research?**

| **Chief Investigator** | **Student Researcher** |
| --- | --- |
| Neil Harris, PhD | Lieke Vorage, Bsc |
| Senior Researcher Public Health School of Medicine Griffith University Gold Coast Campus | Intern, MSc student Maastricht University, the Netherlands  Public Health School of Medicine Griffith University  Gold Coast Campus |
| (07) 555 2787 | (07) 555 27903 |
| [n.harris@griffith.edu.au](mailto:n.harris@griffith.edu.au) | [l.vorage@griffith.edu.au](mailto:l.vorage@griffith.edu.au) |

**What is the purpose of this research?**

The purpose of this research is to understand the motives underlying the attitude towards and consumption intention of functional foods. Part of this research is also dedicated to evaluating the brand image of the functional food company Morlife. This study will be conducted in association with Morlife.

**Your consent to participate**

We ask for your consent to participate in this study; this is indicated by your completion of the survey.

**What you will be asked to do?**

Your participation will involve the completion of a short paper based survey comprising a set of questions with response categories and several open-ended questions. The questions are related to motives underlying the attitude towards and consumption intention of functional foods and the brand image of the functional food company Morlife. The questionnaire will take approximately 10 minutes to complete.

**The expected benefits of the research to Morlife and to participants**

This research forms a component of the student researchers academic program. The results of this research will be included in the student researcher’s thesis and will also be reported to Morlife. Through this research, Morlife will benefit from consumer insights about motives underlying the attitude towards and consumption intention of functional foods and through information about Morlife’s brand image. Participants will get a free food product from Morlife, to thank them for completing the survey.

**Risks to you**

There are no foreseeable risks to you associated with participation in the project. Information will be kept confidential and will not be revealed to other parties.

**Your confidentiality**

The conduct of this research involves the collection, access and/ or use of your identified personal information. The information collected is confidential and will not be disclosed to third parties without your consent, except to meet government, legal or other regulatory authority requirements. A de-identified copy of this data may be used for other research purposes. However, your anonymity will at all times be safeguarded. For further information consult the University's Privacy Plan at [http://www.griffith.edu.au/about-griffith/plans-](http://www.griffith.edu.au/about-griffith/plans-publications/griffith-university-privacy-plan) [publications/griffith-university-privacy-plan](http://www.griffith.edu.au/about-griffith/plans-publications/griffith-university-privacy-plan) or telephone (07) 3735 4375.

All research data (survey responses and analysis) will be retained in a locked cabinet and/or a password protected electronic file at Griffith University for a period of five years before being destroyed. Only members of the research team will have access to the research data.

**Your participation is voluntary**

Participation is voluntary and will in no way impact your relationship with Griffith University or Morlife. You may withdraw at any time without comment or penalty.

**Questions / further information**

For additional information about the project, please contact a member of the research team. You can email either [l.vorage@griffith.edu.au,](mailto:l.vorage@griffith.edu.au) [n.harris@griffith.edu.au,](mailto:n.harris@griffith.edu.au) or contact the School of Medicine on (07) 5552 7869.

**The ethical conduct of this research**

Griffith University conducts research in accordance with the National Statement on Ethical Conduct in Human Research. If you have any concerns about the ethical conduct of the research, you should contact the Manager Research Ethics on (07) 3735 4375 or research- [ethics@griffith.edu.au.](mailto:ethics@griffith.edu.au) The GU reference number for this project is: 2017/166

**Feedback to you**

A one-page summary report on the project findings will be posted at Griffith University Gold Coast Campus, on the notice board in the G01 building, level 3. In addition, a more detailed report will be available to you upon request.

**CONSENT FORM**

I confirm that I have read and understood the information package and in particular have noted that

- I understand that my involvement in this research will include the completion of a short paper based survey
- The questionnaire will take approximately 10 minutes to complete;
- I understand that I am free to withdraw at any time, without comment or penalty;
- I have had any questions answered to my satisfaction;
- I understand the risks involved;
- I understand that my participation in this research is voluntary;
- I understand that there will be no direct benefit to me from participation.

**Consent to participate in the study is indicated by your completion of the survey.**

PLEASE TEAR OFF AND RETAIN FOR YOUR INFORMATION

**QUESTIONNAIRE:** Understanding the motives underlying consumer’s attitude towards and consumption intention of functional foods and evaluating the brand image of the functional food company Morlife

**Please read every question of this survey carefully and answer honestly.**

**Section I: Personal information**

1. **Age**:

***If you are not between the ages 17 and 29 you can return the questionnaire. Thank you for your contribution.**

1. **Gender**:

- Female
- Male

1. **Are you a student at Griffith University?**

- Yes
- No

***If you are not a student at Griffith University you can return the questionnaire. Thank you for your contribution.**

1. **Indicate the year of study at university you are currently enrolled in:**

- 1st
- 2nd
- 3th
- 4th
- Other (please specify):

1. **Describe your living situation:**

- Live with parent(s)/grandparent(s)
- Shared accommodation (non-student)
- Shared accommodation (student)
- Own house or apartment
- Other (please specify):

1. **Who is responsible for food shopping in your household?**

- Me
- Me and someone else
- Someone else

1. **Marital Status**:

- Single
- Married/Partnership
- Separated/Divorced

1. **On average, I work hours per week *(only paid employment)***
2. **What is your total personal annual income before tax from all sources (e.g. wages, government benefits, allowance from parents/family etc.)? (Please tick only one answer)**

- $104,000 - or more ($2000 or more/week)
- $83,200 - $103,999 ($1,600-$1,999/week)
- $67,600 - $83,199 ($1300-$1,599/week)
- $52,000 - $67,599 ($1,000-$1,299/week)
- $41,600 - $51,999 ($800-$999/week)
- $31,200 - $41,599 ($600-$799/week)
- $20,800 - $31,199 ($400-$599/week)
- $10,400 - $20,799 ($200 - $399/week)
- $10,399 – or less ($199 or less/week)

**Section II: Motives Underlying the Selection of Food**

1. **Please indicate the importance of the following items related to your food choice ranging from ‘Not at all important’ to ‘Very Important’.**

| **It is important to me that the food I eat on a typical day:** | **Not at all important** | **A little important** | **Moderately important** | **Very important** |
| --- | --- | --- | --- | --- |
| Contains no additives | 1 | 2 | 3 | 4 |
| Is easy to prepare | 1 | 2 | 3 | 4 |
| Is nutritious | 1 | 2 | 3 | 4 |
| Comes from a country I approve of politically | 1 | 2 | 3 | 4 |
| Helps me cope with stress | 1 | 2 | 3 | 4 |
| Is high in fibre and roughage | 1 | 2 | 3 | 4 |
| Is good for my skin/teeth/hair/nails etc. | 1 | 2 | 3 | 4 |
| Has the country of origin clearly marked | 1 | 2 | 3 | 4 |
| Helps me relax | 1 | 2 | 3 | 4 |
| Has been prepared in an environmentally friendly way | 1 | 2 | 3 | 4 |
| Cheers me up | 1 | 2 | 3 | 4 |

| Contains natural ingredients | 1 | 2 | 3 | 4 |
| --- | --- | --- | --- | --- |
| Keeps me healthy | 1 | 2 | 3 | 4 |
| Can be cooked very simply | 1 | 2 | 3 | 4 |
| Has been produced in a way that animals have not experienced pain | 1 | 2 | 3 | 4 |
| Can be bought in shops close to where I live or work | 1 | 2 | 3 | 4 |
| Tastes good | 1 | 2 | 3 | 4 |
| Is what I usually eat | 1 | 2 | 3 | 4 |
| Looks nice | 1 | 2 | 3 | 4 |
| Has a pleasant texture | 1 | 2 | 3 | 4 |
| Is easily available in shops and supermarkets | 1 | 2 | 3 | 4 |
| Contains a lot of vitamins and minerals | 1 | 2 | 3 | 4 |
| Makes me feel good | 1 | 2 | 3 | 4 |
| Contains no artificial ingredients | 1 | 2 | 3 | 4 |
| Is not expensive | 1 | 2 | 3 | 4 |
| Is packaged in an environmentally friendly way | 1 | 2 | 3 | 4 |
| Is good value for money | 1 | 2 | 3 | 4 |
| Is low in calories | 1 | 2 | 3 | 4 |
| Smells nice | 1 | 2 | 3 | 4 |
| Does not compromise your sporting and exercise goals | 1 | 2 | 3 | 4 |
| Is not forbidden in my religion | 1 | 2 | 3 | 4 |
| Is low in fat | 1 | 2 | 3 | 4 |
| Provides enough energy to get through your physical exercise program | 1 | 2 | 3 | 4 |
| Is familiar | 1 | 2 | 3 | 4 |
| Is like the food I ate when I was a child | 1 | 2 | 3 | 4 |
| Takes no time to prepare | 1 | 2 | 3 | 4 |
| Has been produced in a way that animal rights have been respected | 1 | 2 | 3 | 4 |
| Keeps me awake/alert | 1 | 2 | 3 | 4 |
| Has been produced in a way which has not shaken the balance of nature | 1 | 2 | 3 | 4 |
| Is cheap | 1 | 2 | 3 | 4 |
| Is high in protein | 1 | 2 | 3 | 4 |
| Comes from a country in which human rights are not violated | 1 | 2 | 3 | 4 |
| Helps me cope with life | 1 | 2 | 3 | 4 |
| Has been prepared in a way that does not conflict with my political values | 1 | 2 | 3 | 4 |
| Helps me control my weight | 1 | 2 | 3 | 4 |
| Is in harmony with my religious views | 1 | 2 | 3 | 4 |

**Section III: Functional Foods**

**Before moving to the next questions, PLEASE READ the following definition of ‘Functional Foods’.**

**Functional Food Definition:**

**Functional foods are defined as ‘foods that meet consumer needs for general health and wellbeing, and the prevention and management of compromised health**

**conditions’.**

**For the purpose of this study, we exclude fresh fruit and vegetables as functional foods.**

**A few examples of functional foods are:**

- **Muesli with added fibre**
- **A snack bar with added protein**
- **Juice rich in antioxidants**
- **Margarine which can lower cholesterol**
- **A yoghurt drink with probiotics**
- **Dried goji berries (high in antioxidants)**
- **Chia seeds (high in omega 3 and fibre)**

1. **How often do you consume functional foods?**

- Every day
- 5-6 times a week
- 3-4 times a week
- 1-2 times a week
- 2-3 times a month
- Once a month
- 6-11 times a year
- 2-5 times a year
- Once a year
- I do not consume functional foods

**Question for student that consume functional foods**

**Please SKIP question 23 if you do NOT consume functional foods**

1. **Please indicate the importance of the following items related to your functional food choice ranging from ‘Not at all important’ to ‘Very Important’.**

| **It is important to me that the functional food I eat:** | **Not at all important** | **A little important** | **Moderately important** | **Very important** |
| --- | --- | --- | --- | --- |
| Prevents nutrition related diseases | 1 | 2 | 3 | 4 |
| Improves physical performance | 1 | 2 | 3 | 4 |
| Improves mental performance | 1 | 2 | 3 | 4 |
| Maintains general health and well-being | 1 | 2 | 3 | 4 |
| Is easy to prepare | 1 | 2 | 3 | 4 |
| Is easily available in food supermarkets and shops | 1 | 2 | 3 | 4 |

**If there are other factors important to you when consuming functional foods, please specify them here:**

1. **Please rate your attitude towards functional foods**

|  | **Completely disagree** | **Disagree** | **Neither Agree nor Disagree** | **Agree** | **Completely agree** |
| --- | --- | --- | --- | --- | --- |
| Functional foods are safer for health compared to other products | 1 | 2 | 3 | 4 | 5 |
| Functional foods are healthier compared to other products | 1 | 2 | 3 | 4 | 5 |
| Functional foods have a better taste | 1 | 2 | 3 | 4 | 5 |
| Functional foods fit into the natural way of life | 1 | 2 | 3 | 4 | 5 |
| It is wise to buy functional foods | 1 | 2 | 3 | 4 | 5 |
| Functional foods are of higher quality | 1 | 2 | 3 | 4 | 5 |
| Functional foods form a part of my lifestyle | 1 | 2 | 3 | 4 | 5 |
| Functional foods are expensive | 1 | 2 | 3 | 4 | 5 |
| I need functional foods | 1 | 2 | 3 | 4 | 5 |
| I trust the food manufacturers that produce functional foods | 1 | 2 | 3 | 4 | 5 |
| I like to try new food products, such as functional foods | 1 | 2 | 3 | 4 | 5 |
| Functional foods are easily available where I usually purchase food | 1 | 2 | 3 | 4 | 5 |

**This is the end of the survey**

**THANK YOU FOR YOUR TIME AND COOPERATION!**

## 
