# Supplementary material for: The Association of Demographic Characteristics and Food Choice Motives with the Consumption of Functional Foods in Emerging Adults
Source: Nutrients. 2020 Aug 25;12(9):2582. doi: 10.3390/nu12092582 (PMC7551355; doi:10.3390/nu12092582)
Supplement: Supplementary file 1 [file nutrients-12-02582-s001.zip › Supplements/Supplements_Nutrients_Appendix S3_Statistical_output_Lieke_Vorage.docx]

## Appendix S3: Statistical output

**Table 1: Motives underlying the selection of food (Emerging adults)**

|  | **Items** | **Mean** | **SD** |
| --- | --- | --- | --- |
| Natural content | Contains no additives | 2.28 | 0.93 |
|  | Contains natural ingredients | 2.97 | 0.89 |
|  | Contains no artificial ingredients | 2.53 | 0.88 |
| Health | Contains a lot of vitamins and minerals | 3.06 | 0.80 |
|  | Keeps me healthy | 3.42 | 0.71 |
|  | Is nutritious | 3.34 | 0.73 |
|  | Is high in protein | 2.83 | 0.82 |
|  | Is good for my skin/teeth/hair/nails | 2.70 | 0.89 |
|  | Is high in fibre and roughage | 2.52 | 0.87 |
| Mood | Helps me cope with stress | 2.40 | 0.95 |
|  | Helps me cope with life | 2.59 | 0.92 |
|  | Helps me relax | 2.38 | 0.90 |
|  | Keeps me awake/alert | 2.83 | 0.86 |
|  | Cheers me up | 2.73 | 0.93 |
|  | Makes me feel good | 3.10 | 0.81 |
| Convenience | Is easy to prepare | 3.13 | 0.75 |
|  | Can be cooked very simply | 3.09 | 0.81 |
|  | Takes no time to prepare | 2.51 | 0.91 |
|  | Can be bought in shops close to where I live or work | 3.28 | 0.80 |
|  | Is easily available in shops and supermarkets | 3.26 | 0.70 |
| Sensory appeal | Smells nice | 2.93 | 0.86 |
|  | Looks nice | 2.83 | 0.83 |
|  | Has a pleasant texture | 3.06 | 0.76 |
|  | Tastes good | 3.68 | 0.58 |
| Price | Is not expensive | 3.34 | 0.74 |
|  | Is cheap | 3.15 | 0.82 |
|  | Is good value for money | 3.46 | 0.67 |
| Weight Control | Is low in calories | 2.59 | 0.91 |
|  | Helps me control my weight | 2.78 | 0.94 |
|  | Is low in fat | 2.34 | 0.88 |
| Familiarity | Is what I usually eat | 2.89 | 0.85 |
|  | Is familiar | 2.47 | 0.83 |
|  | Is like the food I ate when I was a child | 1.92 | 0.87 |
| Fitness | Does not compromise your sporting and exercise goals | 2.83 | 0.96 |
|  | Provides enough energy to get through your physical  exercise program | 2.91 | 0.86 |
| Ecological Welfare | Has been produced in a way that animals have not experienced pain | 2.79 | 1.02 |
|  | Has been produced in a way that animal rights have been  Respected | 2.80 | 0.99 |
|  | Has been prepared in an environmentally friendly way | 2.66 | 0.93 |
|  | Has been produced in a way which has not shaken the  balance of nature | 2.58 | 0.89 |
|  | Is packaged in an environmentally friendly way | 2.72 | 0.94 |
| Political Values | Comes from a country I approve of politically | 2.04 | 0.98 |

|  | Comes from a country in which human rights are not violated | 2.63 | 1.00 |
| --- | --- | --- | --- |
|  | Has the country of origin clearly marked | 2.21 | 1.05 |
|  | Has been prepared in a way that does not conflict with  my political values | 2.12 | 0.98 |
| Religion | Is not forbidden in my religion | 1.46 | 0.92 |
|  | Is in harmony with my religious views | 1.60 | 0.95 |

**Table 2: Mann-Whitney U test results for motives underlying the selection of food according to gender (Emerging adults)**

| **Motive** | **Mann-**  **Whitney U** | **Z-score** | **p-value** | **Mean rank** | |
| --- | --- | --- | --- | --- | --- |
|  |  |  |  | Females | Males |
| Natural  content | 10789 | -2.368 | **0.018** | 190.24 | 160.89 |
| Convenience | 12514,5 | -0.451 | 0.652 | 181.52 | 187.14 |
| Health | 11941 | -0.986 | 0.324 | 184.27 | 172.10 |
| Political  Values | 11839 | -0.958 | 0.338 | 183.66 | 171.82 |
| Mood | 11374 | -1.305 | 0.192 | 184.74 | 168.5 |
| Ecological  Welfare | 9953,5 | -3.517 | **0.000** | 195.64 | 151.61 |
| Sensory  Appeal | 11619,5 | -1.856 | 0.063 | 191.12 | 168.07 |
| Familiarity | 12750 | -0.292 | 0.770 | 183.05 | 186.69 |
| Price | 12704 | -0.348 | 0.728 | 185.12 | 180.83 |
| Weight  Control | 11157,5 | -2.230 | **0.026** | 191.83 | 164.03 |
| Fitness | 12450 | -0.984 | 0.325 | 182.27 | 194.46 |
| Religious | 11666.5 | -2.104 | **0.035** | 179.39 | 202.45 |

**Table 3: Mann-Whitney U test results for motives underlying the selection of food according to age groups (Emerging adults)**

| **Motive** | **Mann-**  **Whitney U** | **Z-score** | **p-value** | **Mean rank** | |
| --- | --- | --- | --- | --- | --- |
|  |  |  |  | Young emerging  adults (17-20) | Older emerging  adults(21-29) |
| Natural  content | 14420,5 | -1.460 | 0.144 | 176.05 | 192.36 |
| Convenience | 15864.5 | -0.087 | 0.930 | 183,39 | 182,41 |
| Health | 14426 | -1.165 | 0.244 | 175.87 | 188.91 |
| Political  Values | 14019,5 | -1.518 | 0.129 | 175.87 | 188.91 |
| Mood | 14782,5 | -0.643 | 0.520 | 177.69 | 184.91 |
| Ecological  Welfare | 15885 | -0.173 | 0.863 | 183.23 | 185.19 |
| Sensory  Appeal | 15655 | -0.592 | 0.554 | 182.39 | 189.03 |

| Familiarity | 16013 | -0.044 | 0.965 | 184.19 | 183.70 |
| --- | --- | --- | --- | --- | --- |
| Price | 15789 | -0.275 | 0.783 | 185.20 | 182.15 |
| Weight  Control | 14057.5 | -2.099 | **0.036** | 193.74 | 170.12 |
| Fitness | 15827.5 | -0.492 | 0.623 | 187.66 | 182.16 |
| Religious | 16233.5 | -0.090 | 0.928 | 185.15 | 186.04 |

**Table 4: Mann-Whitney U test results for motives underlying the selection of food according to marital status (Emerging adults)**

| **Motive** | **Mann-**  **Whitney U** | **Z-score** | **p-value** | **Mean rank** | |
| --- | --- | --- | --- | --- | --- |
|  |  |  |  | Single | In a Partnership/  Married/Divorced |
| Natural  content | 9732 | -1.528 | 0.127 | 186.18 | 165.38 |
| Convenience | 10221.5 | -0.366 | 0.715 | 181.51 | 186.53 |
| Health | 10249.5 | -0.151 | 0.880 | 180.91 | 178.85 |
| Political  Values | 9818.5 | -0.787 | 0.431 | 182.17 | 171.50 |
| Mood | 9473.5 | -1.094 | 0.274 | 182.99 | 168.08 |
| Ecological  Welfare | 10509 | -0.230 | 0.818 | 184.13 | 180.96 |
| Sensory  Appeal | 10473 | -0.366 | 0.714 | 185.50 | 180.47 |
| Familiarity | 9659.5 | -1.295 | 0.195 | 187.03 | 169.32 |
| Price | 10578.5 | -0.007 | 0.994 | 183.52 | 183.42 |
| Weight  Control | 10315 | -0.518 | 0.605 | 185.41 | 178.30 |
| Fitness | 10585.5 | -0.272 | 0.785 | 184.26 | 187.99 |
| Religious | 8834 | -2.778 | **0.005** | 191.66 | 158.01 |

**Table 5: Mann-Whitney U test results for motives underlying the selection of food according to living situation (Emerging adults)**

| **Motive** | **Mann-**  **Whitney U** | **Z-score** | **p-value** | **Mean rank** | |
| --- | --- | --- | --- | --- | --- |
|  |  |  |  | Living  dependently | Living  independently |
| Natural  content | 13697 | -1.845 | 0.65 | 169.99 | 190.19 |
| Convenience | 14713.5 | -0.849 | 0.396 | 175.08 | 184.41 |
| Health | 12993 | -2.311 | **0.021** | 166.38 | 191.71 |
| Political  Values | 14144 | -0.985 | 0.325 | 172.00 | 182.78 |
| Mood | 14720 | -0.339 | 0.734 | 178.04 | 174.32 |
| Ecological  Welfare | 15310 | -0.378 | 0.706 | 181.75 | 177.57 |
| Sensory  Appeal | 15467.5 | -0.401 | 0.688 | 179.15 | 183.57 |

| Familiarity | 14816.5 | -0.926 | 0.355 | 175.73 | 185.88 |
| --- | --- | --- | --- | --- | --- |
| Price | 14537 | -1.168 | 0.243 | 174.72 | 187.44 |
| Weight  Control | 14810 | -0.945 | 0.345 | 176.19 | 186.60 |
| Fitness | 15449.5 | -0.499 | 0.618 | 179.22 | 184.69 |
| Religious | 15389.5 | -0.631 | 0.528 | 178.94 | 185.08 |

**Table 6: Mann-Whitney U test results for motives underlying the selection of food according to income (Emerging adults)**

| **Motive** | **Mann-**  **Whitney U** | **Z-score** | **p-value** | **Mean rank** | |
| --- | --- | --- | --- | --- | --- |
|  |  |  |  | Low income  (<20.799) | High income  (>20.800). |
| Natural  content | 10865.5 | -0.967 | 0.334 | 174.00 | 186.03 |
| Convenience | 10975,5 | -0.984 | 0.325 | 174.42 | 186.68 |
| Health | 10529,5 | -1.271 | 0.204 | 172.04 | 187.85 |
| Political  Values | 10769,5 | -0.978 | 0.328 | 172.95 | 185.12 |
| Mood | 11205,5 | -0.236 | 0.814 | 174.27 | 177.20 |
| Ecological  Welfare | 11647,5 | -0.279 | 0.780 | 177.62 | 182.13 |
| Sensory  Appeal | 10791 | -1.512 | 0.131 | 174.76 | 193.60 |
| Familiarity | 11166 | -0.964 | 0.335 | 175.48 | 187.43 |
| Price | 10809,5 | -1.303 | 0.193 | 182.51 | 166.46 |
| Weight  Control | 11664 | -0.314 | 0.754 | 178.59 | 184.21 |
| Fitness | 11726 | -0.453 | 0.651 | 178.59 | 184.21 |
| Religious | 15361 | -1.134 | 0.257 | 183.12 | 170.68 |

**Table 7: Attitudes toward functional food, mean and SD (Emerging adults)**

| **Scale** | **Items** | **Mean** | **SD** | **Cronbach’s α** |
| --- | --- | --- | --- | --- |
| Attitudes toward  functional food |  | 3.52 | 0.59 | 0.835 |
|  | Functional foods are safer for health compared to other  products | 3.62 | 0.77 |  |
|  | Functional foods are healthier compared  to other products | 3.71 | 0.80 |  |
|  | Functional foods  have a better taste | 3.11 | 0.77 |  |
|  | Functional foods fit into the natural way  of life | 3.56 | 0.81 |  |
|  | It is wise to buy  functional foods | 3.68 | 0.79 |  |
|  | Functional foods are  of higher quality | 3.53 | 0.81 |  |
|  | Functional foods form a part of my  lifestyle | 3.45 | 3.45 |  |

**Table 8: Mann-Whitney U test results for attitudes toward functional food according to gender, age, living situation and income (Emerging adults)**

| **Attitudes** | **Mann-**  **Whitney U** | **Z-score** | **p-value** | **Mean rank** | |
| --- | --- | --- | --- | --- | --- |
|  |  |  |  | Females | Males |
| Gender | 12027 | -1.296 | 0.195 | 189.28 | 172.99 |
|  |  |  |  | Younger emerging  Adults | Older emerging adults |
| Age | 12768 | -3.481 | **0.001** | 200.50 | 161.06 |
|  |  |  |  | Low income | High income |
| Income | 12037 | -0.027 | 0.978 | 179.41 | 179.76 |
|  |  |  |  | Living  dependent | Living  independent |
| Living  situation | 14573 | -1.315 | 0.189 | 187.10 | 172.51 |
|  |  |  |  | Single | Married/Divorced |
| Marital Status | 10218 | -0.667 | 0.498 | 186.36 | 176.97 |

**Table 9: Functional food consumption (Emerging adults)**

| **Categories** | **Frequency** | |
| --- | --- | --- |
|  | % | N |
| Every day | 31.6 | 117 |
| 5-6 times a week | 14.1 | 52 |
| 3-4 times a week | 21.4 | 79 |
| 1-2 times a week | 19.5 | 72 |
| 2-3 times a month | 6.8 | 25 |
| Once a month | 2.7 | 10 |
| 6-11 times a year | 2.2 | 8 |
| 2-5 times a year | 1.1 | 4 |
| Once a year | 0.5 | 2 |
| I do not consume functional  foods | 0.3 | 1 |

**Table 10: Mann-Whitney U test results for functional food consumption according to gender, age, income, living situation and marital status (Emerging adults)**

| **Functional food**  **consumption** | **Mann- Whitney U** | **Z-score** | **p-value** | **Mean rank** | |
| --- | --- | --- | --- | --- | --- |
|  |  |  |  | Females | Males |
| Gender | 11916 | -1.597 | 0.110 | 180.31 | 199.91 |
|  |  |  |  | Younger emerging  adults | Older emerging adults |
| Age | 15158 | -1.181 | 0.238 | 190.63 | 177.54 |
|  |  |  |  | Low income | High income |
| Income | 11835 | -0.325 | 0.745 | 179 | 183 |
|  |  |  |  | Living  dependent | Living  independent |
| Living  situation | 13510.5 | -2.531 | **0.011** | 192.97 | 165.47 |
|  |  |  |  | Single | Married/Divorced |
| Marital status | 8.882 | -2.418 | **0.016** | 191.49 | 158.67 |

**Table 11: Correlation between food choice motives and attitudes (Emerging adults)**

| **Food choice motives** | **Attitude towards functional food** | |
| --- | --- | --- |
|  | Spearman rho | p-value |
| Natural content | 0.212 | **0.000** |
| Convenience | 0.052 | 0.323 |
| Health | 0.226 | **0.000** |
| Political Values | 0.145 | **0.006** |
| Mood | 0.187 | **0.000** |
| Ecological Welfare | 0.247 | **0.000** |
| Sensory Appeal | 0.061 | 0.247 |
| Familiarity | 0.137 | **0.009** |
| Price | -0.009 | 0.865 |
| Weight Control | 0.314 | **0.000** |
| Fitness | 0.207 | **0.000** |
| Religious | 0.138 | **0.008** |

**Table 12: Correlation between food choice motives and functional food consumption (Emerging adults)**

| **Food choice motives** | **Functional Food Consumption** | |
| --- | --- | --- |
|  | Spearman rho | p-value |
| Natural content | 0.184 | **0.000** |
| Convenience | 0.014 | 0.792 |
| Health | 0.253 | **0.000** |
| Political Values | 0.089 | 0.092 |
| Mood | 0.071 | 0.179 |
| Ecological Welfare | 0.138 | **0.008** |
| Sensory Appeal | -0.014 | 0.786 |
| Familiarity | -0.013 | 0.801 |
| Price | -0.030 | 0.565 |
| Weight Control | 0.162 | **0.002** |
| Fitness | 0.245 | **0.000** |
| Religious | -0.006 | 0.903 |
