# Supplementary material for: The Association of Demographic Characteristics and Food Choice Motives with the Consumption of Functional Foods in Emerging Adults
Source: Nutrients. 2020 Aug 25;12(9):2582. doi: 10.3390/nu12092582 (PMC7551355; doi:10.3390/nu12092582)
Supplement: Supplementary file 1 [file nutrients-12-02582-s001.zip › Supplements/Supplements_Nutrients_Appendix_S2_Recoding_Lieke_Vorage.docx]

## Appendix S2: Recoding

**Table 1: Recoding demographic variables (Emerging adults)**

| **Variable** | **Original categories** | **Recoded categories** |
| --- | --- | --- |
| Age | 1. years 2. years 3. years 4. years 5. years 6. years 7. years 8. years 9. years 10. years 11. years 12. years 13. years | Younger group  (17 to 20 years old)  Older group  (21 to 29 years old) |
| Marital status | Single Married/Partnership Separated/Divorced | Single Married/Divorced |
| Living situation | Live with parent(s)/parent(s) Shared accommodation (student)  Shared accommodation (non- student)  Own house or apartment Other | Living dependently Living independently |
| Income | $10,399 – or less ($199 or less/week)  $10,400 - $20,799 ($200 -  $399/week)  $20,800 - $31,199 ($400-  $599/week)  $31,200 - $41,599 ($600-  $799/week)  $41,600 - $51,999 ($800-  $999/week)  $52,000 - $67,599 ($1,000-  $1,299/week)  $67,600 - $83,199 ($1300-  $1,599/week)  $83,200 - $103,999 ($1,600-  $1,999/week)  $104,000 - or more ($2000 or more/week) | ≤$20.799  >$20.800 |

**Table 2: Recoding binary logistic regression**

| **Variable** | **Original categories** | **Recoded categories** |
| --- | --- | --- |
| Natural Content | Ranging from 1 (Not at all important) to 4 (Very important) | 1.00-2.67 (Low score natural content) 2.68-4.00 (High score on natural content) |
| Health | Ranging from 1 (Not at all  important) to 4 (Very important) | 1.00-3.00 (Low score health)  3.01-4.00 (High score on health) |
| Political values | Ranging from 1 (Not at all important) to 4 (Very important) | 1.00-2.25 (Low score political values) 2.26-4.00 (High score political values) |
| Mood | Ranging from 1 (Not at all important) to 4 (Very important) | 1.00-2.75 (Low score mood) 2.76-4.00 (High score mood) |
| Ecological welfare | Ranging from 1 (Not at all important) to 4 (Very important) | 1.00-2.80 (Low score on ecological welfare)  2.81-3.00 (High score on ecological welfare |
| Familiarity | Ranging from 1 (Not at all  important) to 4 (Very important) | 1.00-2.33 (Low score on familiarity)  2.34-4.00 (High score on familiarity) |
| Weight control | Ranging from 1 (Not at all  important) to 4 (Very important) | 1.00-2.67 (Low score on weight control)  2.68-4.00 (High score on weight control) |
| Fitness | Ranging from 1 (Not at all  important) to 4 (Very important) | 1.00-3.00 (Low score on fitness)  3.01-4.00 (High score on fitness) |
| Religion | Ranging from 1 (Not at all  important) to 4 (Very important) | 1.00-1.09 (Low score on religion)  1.10-4.00 (High score on religion) |
| Attitude towards  functional food | Ranging from 1 (Completely  disagree) to 5 (Completely agree) | 1.00-3.43 (Negative attitude)  3.44-5.00 (Positive attitude) |
| Functional food consumption | 1. Every day 2. 5-6 times a week 3. 3-4 times a week 4. 1-2 times a week 5. 2-3 times a month 6. Once a month 7. 6-11 times a year 8. 2-5 times a year 9. Once a year 10. I do not consume functional   foods | 1-3 (High consumption of functional food)  4-10 (Low consumption of functional food) |

## 
